# Editorial for the Special Issue “Adaptation, Aging, and Cell Death in Yeast Stress Response: Models, Mechanisms and Applications”

**DOI:** 10.3390/microorganisms10061126

**Published:** 2022-05-30

**Authors:** Nicoletta Guaragnella, Anita Krisko, Tiago F. Outeiro

**Affiliations:** 1 Department of Biosciences, Biotechnology and Biopharmaceutics, University of Bari “Aldo Moro”, 70125 Bari, Italy; 2 Department of Experimental Neurodegeneration, University Medical Center Goettingen, Waldweg 33, 37073 Göttingen, Germany; anita.krisko@med.uni-goettingen.de (A.K.); tiago.outeiro@med.uni-goettingen.de (T.F.O.); 3 Max Planck Institute for Natural Sciences, 37075 Göttingen, Germany; 4 Translational and Clinical Research Institute, Faculty of Medical Sciences, Newcastle University, Framlington Place, Newcastle Upon Tyne NE2 4HH, UK; 5 Scientific Employee with an Honorary Contract at Deutsches Zentrum für Neurodegenerative Erkrankungen (DZNE), 37075 Göttingen, Germany

Every cell experiences different types of stress during its life cycle. The capacity of each cell to counteract stress makes the difference in terms of adaptation, aging and, ultimately, cell death. Specific stress responses depend on intrinsic and extrinsic factors related to multiple aspects: the duration of stress exposure, i.e., transient or chronic, the stressor’s concentration and the rate threshold, the cell growth state, i.e., dividing versus non-dividing cells, and the surrounding environment. Most of the stress conditions can be lethal if severe or prolonged; however, when transient and/or mild, they promote adaptive responses. Such a dose response of the cell/organismal survival is called hormesis [1]. Hormetic responses have so far been observed to extend lifespan in budding yeast, nematodes, fruit fly and rodents. Even though the mechanisms employed by different stressors may differ, the unifying feature of hormetic responses is the activation of maintenance and repair machineries [2].

The budding yeast *Saccharomyces cerevisiae* is an invaluable model organism for studying the molecular mechanisms underlying stress responses and regulation of cell fate. The knowledge gained using yeast cells, together with the evolutionary conservation of genes, proteins, and pathways, represents a valuable asset for studies in other relevant systems, ultimately enabling the translation to humans.

The goal of this Special Issue is to provide a wide cellular perspective on different stress conditions by highlighting the complexity of the responses starting from a simple eukaryotic organism. The collection includes eight contributions, including seven research articles and one review, in which different aspects are explored: the stress impact on physiological processes; common or specific upstream and downstream mechanisms; and the involvement of subcellular components/organelles, stress signaling pathways and their interplay in the response. 

Two articles deal with cell challenge to osmotic and salt stress. In the article “The Effect of Lithium on the Budding Yeast Saccharomyces cerevisiae upon Stress Adaptation”, the authors studied the effect of lithium chloride on yeast cell growth and viability, on protein aggregate formation and on cell volume. Lithium treatment has been shown to extend the lifespan of several organisms and promote recovery following stress [3,4]. The convergence of general and specific stress response emerge from the data presented, with the significance of nutritional environment on cell stress sensitivity and the role of stress-specific master regulators, such as glucose starvation and Hog1 in this case. 

The article “RTG Signaling Sustains Mitochondrial Respiratory Capacity in HOG1-Dependent Osmoadaptation” is focused on the role of the RTG-dependent retrograde signaling in salt-induced osmotic stress and its interplay with *HOG1* [5,6]. The data presented highlight the spatial and temporal interplay between stress signaling pathways, with short- and long-term responses and inter-organellar metabolic cross talk, in particular peroxisomes–mitochondria–nucleus in osmoadaptation. 

The decline in physiological functions due to aging is part of cellular demise and involves individual stress response capacity. The article “The Role of Sch9 and the V-ATPase in the Adaptation Response to Acetic Acid and the Consequences for Growth and Chronological Lifespan” analyzes the involvement of the protein kinase Sch9 and the Vacuolar ATPase in the Adaptive Response to Acetic Acid and the related consequences on Growth and Chronological Lifespan. Molecular mechanisms underlying acetic acid stress response in yeast have been characterized in detail [7,8]. This study emphasizes the relevance of the temporal dynamics in stress response: shorter and faster, as a primary line of defense, and later for long-term adaptation. In addition, the role of lipid metabolism in integrating stress response, adaptation and lifespan is highlighted. Interestingly, a presumable example of hormesis effect is reported in this contribution, where the stressor is shown to exert cytoprotection, extending longevity. 

Many lines of evidence point to a role of epigenetic modifications in stress response, cell death and senescence [9]. The article “Epigenetic Response of Yarrowia lipolytica to Stress: Tracking Methylation Level and Search for Methylation Patterns via Whole-Genome Sequencing” reports the global DNA methylation status under heat stress conditions in the industrially relevant yeast species, *Yarrowia lipolytica*. Although the authors did not observe differences in the epigenetic response due to stress conditions, the study reveals that the growth phase itself affects the methylation pattern profile, suggesting a more relevant role of epigenetics in cell physiology rather than in heat stress response in this non-conventional yeast species. Amino acid metabolism has drawn much attention due to its importance in lifespan extension [10]. The article ‘’Longevity Regulation by Proline Oxidation in Yeast’’ reports that proline oxidase Put1 regulates chronological lifespan in budding yeast. Supplementation of the medium with proline was able to extend the lifespan only in wild-type yeast, but not in the absence of Put1. The detailed mechanism remains unknown. However, proline oxidation was shown to contribute to the maintenance of the mitochondrial membrane potential and ATP production, emphasizing its importance in the aging process.

The formation of non-membrane-bound compartments in cells has been in the spotlight for decades due to its involvement in diseases and aging [11]. In the article entitled “Neuroserpin Inclusion Bodies in a FENIB Yeast Model”, the authors demonstrate that yeast can be a valuable model for studying human diseases. Familial encephalopathy with neuroserpin inclusion bodies (FENIB) is caused by a point mutation in *SERPINI1* gene, leading to the segregation of neuroserpin 1 (NS) into inclusion bodies. In yeast, both wild-type and mutant NS localize to the ER, delay the exit from the lag phase and in general recapitulate cellular phenotypes observed previously in the mammalian cells. 

The regulation of iron storage and detoxification is an important issue across species [12]. In yeast, this process is regulated by the Ccc1 transporter. In the article ‘’ An Internal Promoter Drives the Expression of a Truncated Form of *CCC1* Capable of Protecting Yeast from Iron Toxicity’’, the authors describe the structure of the transcriptional regulation of the *CCC1* gene. They have found that one of the regulatory regions is located within the coding sequence, driving the expression of the N-terminal truncation version of the transporter. The truncated version of the protein is, like the wild type, able to promote iron accumulation in the vacuole, thus protecting the cells from iron toxicity. The conditions in which this version of Ccc1 is expressed remain to be found. 

Last but not least, the issue includes the review article ‘’Cdk8 Kinase Module: A Mediator of Life and Death Decisions in Times of Stress’’. In yeast, Cdk8 Kinase Module (CKM) negatively regulates a subset of stress-responsive genes (SRGs), with downstream effects on mitochondrial morphology, ubiquitin–proteasome system, and autophagy, thus impacting decisions on cellular death or survival. 

As a whole, the articles in this Special Issue illustrate the complexity of selected stress responses and the signaling pathways that regulate cell fate in the simple eukaryote budding yeast. Our goal was to lay the foundation for our understanding of the molecular underpinnings of stress, aging and cell death, essential aspects of a variety of human conditions that require urgent attention and the development of novel therapeutic strategies.
